# Early development of rostrum saw-teeth in a fossil ray tests classical theories of the evolution of vertebrate dentitions

**DOI:** 10.1098/rspb.2015.1628

**Published:** 2015-10-07

**Authors:** Moya Meredith Smith, Alex Riley, Gareth J. Fraser, Charlie Underwood, Monique Welten, Jürgen Kriwet, Cathrin Pfaff, Zerina Johanson

**Affiliations:** 1 Department of Earth Sciences, Natural History Museum, London SW75BD, UK; 2 Dental Institute, Craniofacial Development, King's College London, London SE1 9RT, UK; 3 Department of Animal and Plant Sciences, University of Sheffield, Sheffield S10 2TN, UK; 4 Department of Earth and Planetary Sciences, Birkbeck, University of London, London WC1E 7HX, UK; 5 Department of Palaeontology, University of Vienna, Vienna 1090, Austria

**Keywords:** Cretaceous ray, tooth regeneration, developmental model, convergent evolution, tooth development

## Abstract

In classical theory, teeth of vertebrate dentitions evolved from co-option of external skin denticles into the oral cavity. This hypothesis predicts that ordered tooth arrangement and regulated replacement in the oral dentition were also derived from skin denticles. The fossil batoid ray *Schizorhiza stromeri* (Chondrichthyes; Cretaceous) provides a test of this theory. *Schizorhiza* preserves an extended cartilaginous rostrum with closely spaced, alternating saw-teeth, different from sawfish and sawsharks today. Multiple replacement teeth reveal unique new data from micro-CT scanning, showing how the ‘cone-in-cone’ series of ordered saw-teeth sets arrange themselves developmentally, to become enclosed by the roots of pre-existing saw-teeth. At the rostrum tip, newly developing saw-teeth are present, as mineralized crown tips within a vascular, cartilaginous furrow; these reorient via two 90° rotations then relocate laterally between previously formed roots. Saw-tooth replacement slows mid-rostrum where fewer saw-teeth are regenerated. These exceptional developmental data reveal regulated order for serial self-renewal, maintaining the saw edge with ever-increasing saw-tooth size. This mimics tooth replacement in chondrichthyans, but differs in the crown reorientation and their enclosure directly between roots of predecessor saw-teeth. *Schizorhiza* saw-tooth development is decoupled from the jaw teeth and their replacement, dependent on a dental lamina. This highly specialized rostral saw, derived from diversification of skin denticles, is distinct from the dentition and demonstrates the potential developmental plasticity of skin denticles.

## 1. Introduction

An evolutionary and developmental link between external skin denticles and the oral dentition remains controversial [1–3]. This link is suggested by the classical theory that in vertebrate evolution, oral teeth were derived from the dermal skin denticles (placoid scales in chondrichthyans). Consequently, teeth and skin denticles should share a common development (for example, similarities in gene expression [4,5]). These similarities in the individual developmental module (tooth or denticle) should extend to their ordered patterning and replacement, two fundamental features of the functional oral dentition [3]. As potential examples of this process, elongate cartilaginous rostra with ‘saw-teeth’ along their edges have evolved within both major chondrichthyan crown groups: the Holocephali [6] and Elasmobranchii (e.g. sawfish, sawsharks, extinct sclerorhynchid rays [7]). Of particular interest is the Cretaceous sclerorhynchid ray *Schizorhiza stromeri* Weiler in Stromer & Weiler 1930, possessing an extended rostrum with saw-teeth in a close-packed and distinct alternate pattern [8], differing strongly from saw-teeth along extant sawfish and sawshark rostra, but similar to the alternating pattern characteristic of the chondrichthyan dentition. In both the sawfish and sawsharks, rostrum saw-teeth are regularly spaced along the rostrum, and replacement saw-teeth only form in sawsharks after the existing saw-tooth is lost, while in sawfish they are not replaced, but instead each one grows continuously [9,10]. Saw-teeth in these taxa are distinct from the oral dentition and show more similarities in development and replacement to external skin denticles [10].

With saw-teeth putatively more similar to the oral dentition, *Schizorhiza* provides a tractable model to further test the hypothesis for the evolution of teeth that links dermal denticles and oral dentitions. We investigated sequential developmental stages for saw-tooth replacement, stacked ‘cone within cone’ beneath each functional saw-tooth [8], an arrangement approaching structural patterning in oral dentitions with multiple replacement teeth (110–120 organized saw-tooth files [8]). However, important differences in this replacement relative to chondrichthyan oral teeth suggest that *Schizorhiza* saw-teeth represent modified denticles. We suggest that this extinct taxon models complex ‘tooth’ replacement outside the mouth as an example of diversification of skin denticles but is decoupled from the evolution of oral dentitions and the dental lamina-driven replacement system in the jaws.

## 2. Material and methods

Specimens of rostra of *S. stromeri* were obtained from commercial sources from Maastrichtian (Cretaceous) age phosphorites near Oued Zem, Morocco, but without detailed provenance. These include an articulated partial rostrum tip (NHMUK PV P.73626), two more proximal (towards the chondrocranium) articulated portions of rostrum (NHMUK PV P.73625, Naturhistorisches Museum in Wien Inv.NR 1999z009/0001a), and a near-complete and largely articulated rostrum (NHMUK PV P.73625). Numerous isolated rostral saw-teeth were also collected from a number of sites across Morocco (CJU). Specimens (except for the large articulated rostrum) were scanned using a Metris X-Tek HMX ST 225 CT scanner (Imaging and Analysis Centre, Natural History Museum, London), GE Locus SP CT Tech scanner (KCL, London), Viscom X8060 (Department of Anthropology, University of Vienna; 160 kV, 300 mA, time 1400 ms, filter, 1 mm copper). Three-dimensional renderings, segmentation and analyses were performed using Avizo Standard v. 8.1 (http://www.vsg3d.com/avizo/standard), VG Studio Max v. 2.0 (http://www.volumegraphics.com/en/products/vgstudio-max.html) and Drishti v. 3.02 (http://sf.anu.edu.au/Vizlab/drishti). Due to a high prevalence of broken roots, we used Aviso segmentation tool in our primary morphometric analysis; calculating saw-tooth cap volumes (from apex to widest coronal point; figure 3*a*, inset) for direct comparison between NHMUK PV P.73626, P.73627 and Naturhistorisches Museum in Wien Inv.NR 1999z009/0001a, as well as quantifying saw-tooth disparity in NHMUK PV P.73626. In these, saw-teeth constituting the functional saw were selected as ones most likely to have completed morphogenesis. In conjunction with volumetric calculations, we measured saw-tooth height (from apex to the end of medial root lobe) manually using callipers (also in NHMUK PV P.73625). We used R v. 3.1.2 and RStudio v. 0.98.1091 for statistical analyses (one-way ANOVA and Tukey multiple means comparisons test; cap volumes were log transformed due to unequal variances) and for graphical representations of data. Schematic drawings in figure 4 were created using Pixelmator v. 1.1. A portion preserving saw-tooth files was removed and embedded for sectioning. Sections (NHMUK PV P.73626) were 60 µm thick mounted and covered on glass slides. Photographs were either taken on a Leica MZ95, or a Zeiss photomicroscope II with Nikon 100, in both Nomarski and polarized light with a gypsum plate, and processed using PS CS or Leica Application Suite.

## 3. Investigation and observations

*Schizorhiza* occurs rarely in numerous Late Cretaceous deposits along the southern margin of the western Tethys and the western part of the Atlantic Ocean including the Gulf Coastal Plain ranging from the Santonian to Maastrichtian (*ca* 86–66 Ma [11]). The oldest records are from Jordan, while Campanian and Maastrichtian records are from North Africa and North America [8,11], indicating a rapid westward migration across the Atlantic Ocean. Its rare distribution in coastal deposits and across open marine areas, and the size of the almost complete rostrum (NHMUK PV P.73625, electronic supplementary material, figure S1a,b), suggest that *Schizorhiza* was a medium-sized pelagic ray, contrary to the widely held assumption that sclerorhynchids were bottom-dwellers [12].

**Figure 1.**
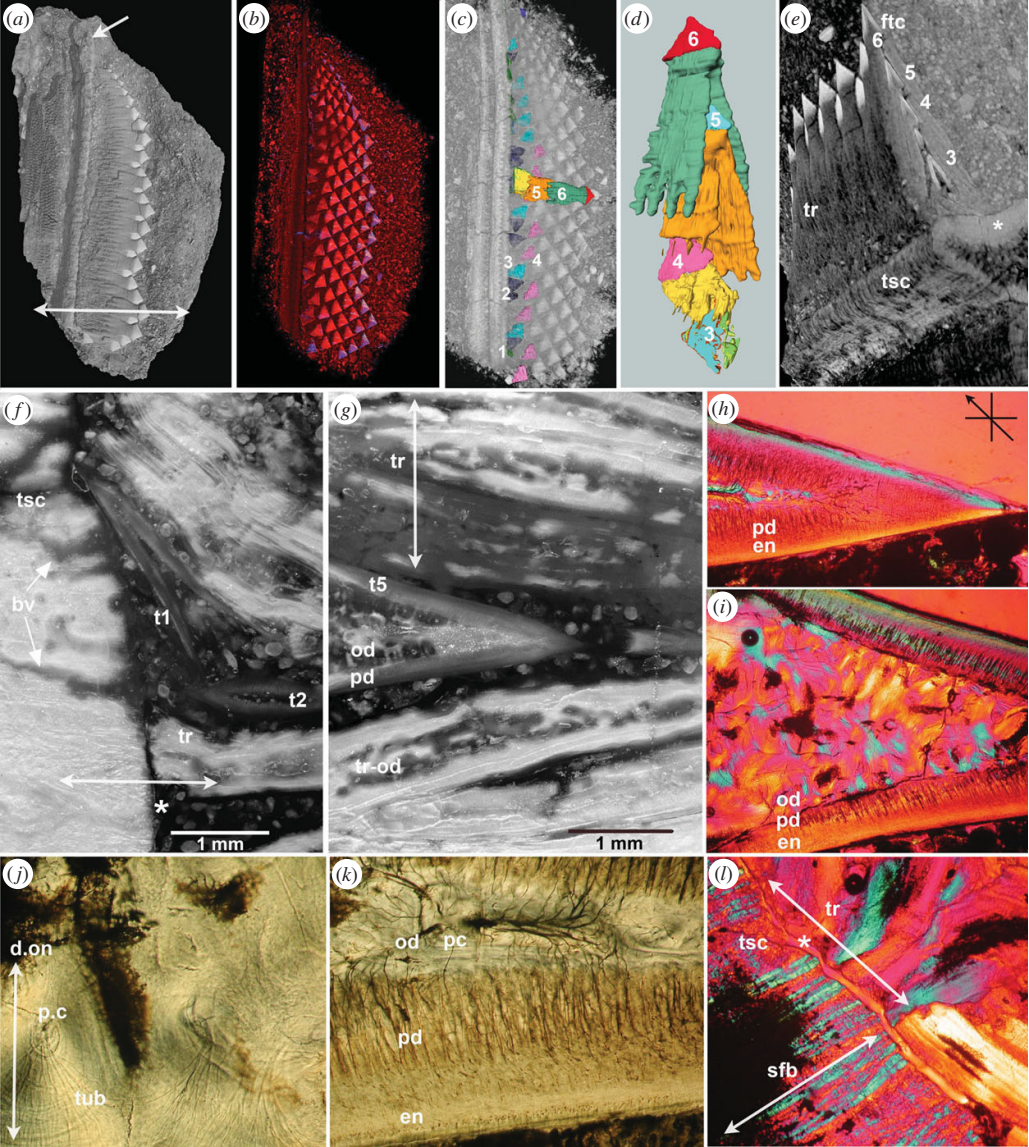
Rostrum with developing saw-teeth, crown and root histology. *Schizorhiza stromeri* Maastrichtian (Latest Cretaceous), Oved Zem region, Morocco. (*a*–*l*) NHMUK PV P.73626, smallest specimen (figure 3). (*a*–*e*) Micro-CT volume-rendered models with segmentation showing arrangement of saw-teeth; size increases sharply from distal to proximal, at a certain point, saw-teeth are of similar size, rostrum tip lacks saw-teeth (arrow; double arrow is section plane in (*f*–*l*)). (*a*) Exposed surface shows wave-like arrangement of arrowhead-shaped, exposed saw-tooth crown. (*b*) Lower-density roots removed virtually, showing stacked crowns extending laterally from cartilage support surface (red to purple, higher density), arranged in close-packed, alternate rows. (*c*) Saw-tooth crowns with developing saw-teeth in rotation phase 1–4; first forming are laterally flattened against the cartilage support furrow ((*e*,*f*) t1); second, dorsoventrally flattened crown tips pointing caudally, then laterally ((*f*) t2), before moving into position within the saw-tooth file and between the roots of older saw-teeth (5, 6, position of segmented tooth set in (*d*)). (*d*) Segmented tooth file showing different positions of saw-tooth crown (3, 4), before moving into position beneath roots of older saw-teeth (5, 6), while roots develop (electronic supplementary material, Video). (*e*) Dorsal surface, plus vertical virtual section through middle of saw-tooth file, saw-tooth crowns with highest mineralization (tsc, mineralized support cartilage; *, developmental furrow), stacked saw-tooth crowns (3–6 of saw-tooth file) aligned between roots (tr) of previous saw-teeth (as in (*c*,*d*)). (*f*–*l*) Tissue composition of saw-teeth (lateral to right). (*f*,*g*) Reflected light; (*f*) attachment of roots to saw-tooth support cartilage, blood vessels (bv) supplying teeth, fibre direction in cartilage and saw-tooth root (double arrow, asterisk, soft tissue junction, see (*L*) gap in +ve birefringent fibres); new saw-tooth crown (t1) flattened laterally onto cartilage, sub-parallel orientation; second rotation saw-tooth (t2) proximo-distally flattened. (*g*) More lateral field, new saw-tooth crown (t5) below roots of all saw-teeth; osteodentine (od) fills in pallial dentine cone (pd) below thin enameloid, and in roots (tr-od). (*h*,*i*,*l*) Polarized light, gypsum plate (arrow 45° to crossed P+A) shows colour of birefringence as blue (+ve) or yellow (−ve) reflecting crystallite orientation (aligned along original collagen fibre bundles) and attachment fibre direction. (*h*) Functional saw-tooth crown tip with very thin enameloid (en), crystallites orthogonal, over pallial dentine (pd). I, field below tip, solid infill of osteodentine with organized osteodentine crystal fibres (+ve and −ve signs of birefringence). (*j*,*k*) Nomarski optics; (*j*) central pulp field with denteone (d.on, double arrow) and tiny pulp canal (pc), from which dentine tubules emerge (tub). (*k*) Field near tip, enameloid, pallial dentine, osteodentine with small pulp canal, fine tubules extend through dentine of both types. (*l*) Attachment region (field at 45° to (*f*)) showing direction of crystal Sharpey's fibre bundles (sfb), many in support cartilage and thick groups in root ends (tr; * gap for soft tissue fibres). Scale bars, *h*,*i*,*l* = 500 µm: *j*,*k* = 100 µm.

**Figure 2.**
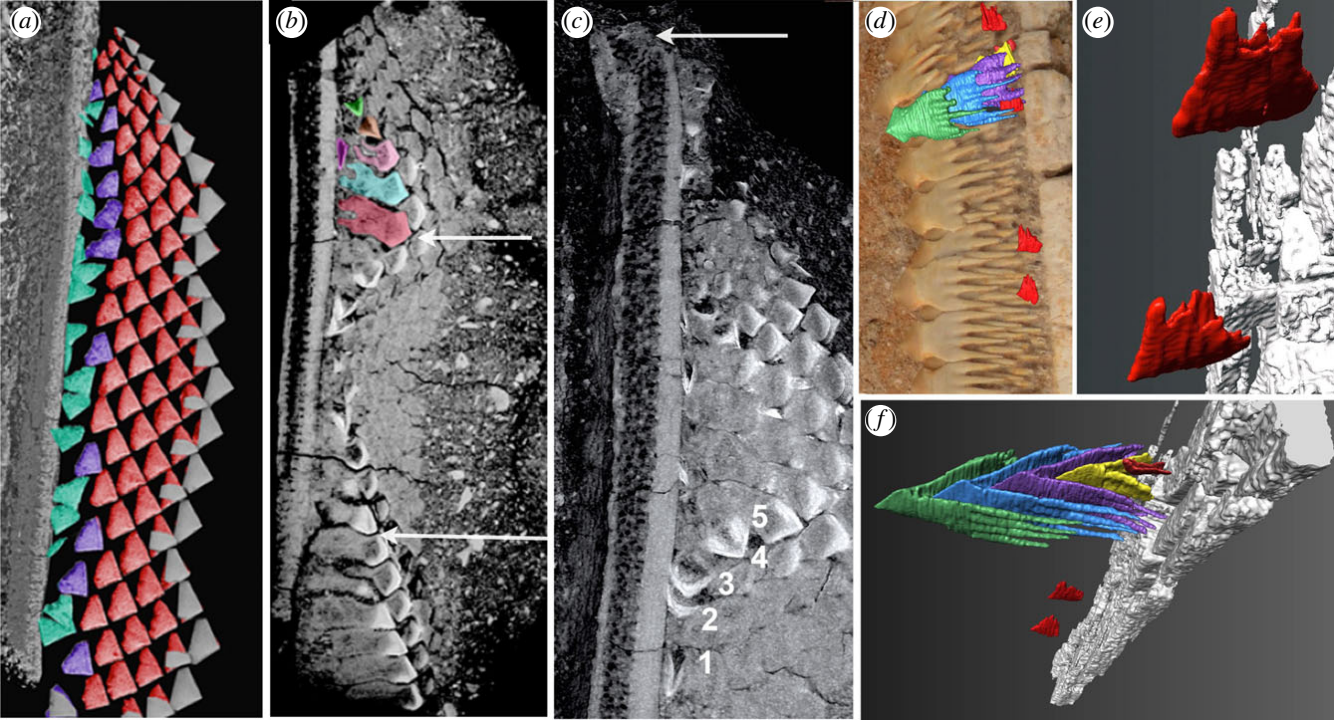
Volume-rendered micro-CT scans showing stages of saw-tooth development. *Schizorhiza stromeri* Maastrichtian (Latest Cretaceous) Oved Zem region, Morocco. (*a*–*c*) NHMUK PV P. 73626, saw-teeth in the distalmost rostrum (figure 1*a*–*c*), distal at top, micro-CT rendered density volume models. Saw-tooth size increases disto-proximally, rostrum tip lacking saw-teeth (arrow, (*c*)), saw-tooth files near tip have the smallest saw-teeth, with progressively more saw-teeth per file, away from the rostrum tip. (*a*) Surface render (dorsoventral), saw-tooth crowns with highest density after roots dissected away, false colour. As saw-teeth develop, crown tips are oriented caudally (green), shifting to partly lateral below root tips (purple), then immediately below root space of older saw-teeth (red), all crowns close-packed in alternate positions, final positions show rostro-caudal wave of saw-tooth crowns (grey). (*b*) Surface render, two groups with timed wave of saw-tooth initiation (arrows), one illustrated in false colours (fuschia, laterally flattened against rostrum; green, dorsoventrally flattened, crown tip caudal; orange, partially rotated; pink, crown tip lateral, roots forming; blue and red, position of alternate tooth files, longer roots). (*c*) Ventrally deeper surface render, showing sets of developing saw-teeth between those in (*b*) smallest developing crowns below saw-tooth roots visible, saw-tooth timed series with individual saw-teeth (1–5) showing rotation into lateral position within the file (5; arrow, toothless rostrum tip). (*d*–*f*) Naturhistorisches Museum in Wien Inv.NR 1999z009/0001a, larger saw-teeth mid-rostrum. (*d*) Macrophoto showing functional tooth row, exposed roots, with superimposed segmented saw-tooth stack and caudally orientated replacement tooth crowns (red) lying below these roots. (*e*) Two hollow segmented developing crowns (red; see green in (*b*)) close to lateral cartilage (grey) showing second stage of orientation during two-stage 90° rotation relative to rostrum. (*f*) Oblique, antero-ventral view of saw-tooth stack (cone in cone, colours red to green for each, successive saw-tooth addition), including positions of a small number of early stage replacement crowns. N.B. different colours (figure 1*c*,*d*) for these mature replacement teeth.

Four articulated partial to near-complete rostra of *Schizorhiza* from the Cretaceous of Morocco were studied, including two showing preserved rostrum tips with functional saw-tooth crowns. Volume-rendered and segmented micro-CT scans, as well as histological thin sections, were examined to investigate saw-tooth arrangement and development. The *Schizorhiza* rostrum comprises a near continuous battery of staggered saw-teeth, arranged laterally to form the functional saw edge. Saw-teeth are absent from the rostrum tip, as well as at the caudal base of the rostrum (figures 1*a* and 2*c*; electronic supplementary material, figure S3a,b, asterisk). Saw-teeth appear abruptly rostral to this base (electronic supplementary material, figure S3a,c, black arrows). Proximo-lateral and rostro-caudal waves of ordered saw-tooth files produce an undulating saw surface (figures 1 and 2*b*,*c*; electronic supplementary material, figures S1a and S3a). Between the functional rostrum saw edge and the rostrum cartilage is a wide zone composed of the stacked saw-tooth roots (figures 1*a* and 2*d*; electronic supplementary material, figures S1 and S3). These four-lobed, bifurcated roots extend towards a densely mineralized, shallow convex supporting cartilage on the rostrum edge, additional to the tessellated surface of the rostrum (figure 1*e*, asterisk, and figure 2*b*,*c*; electronic supplementary material, figure S1c). Developing saw-teeth are not located in sockets but in a shallow groove in this support cartilage (figure 1*c*,*e*; electronic supplementary material, figures S1c and S2a–c). Saw-tooth crowns lie below the functional teeth, fitting between their roots (figures 1*c*–*g* and 2*b*,*f*; electronic supplementary material, figures S1c and S2a,c). A single saw-tooth file shows a developmental series of up to six saw-teeth (figures 1*c*–*e* and 2*f*; electronic supplementary material, figure S1c); across the rostrum saw edge crowns are regularly spaced and display an organization in diagonal rows and horizontal alternating rows (figures 1*a*–*c* and 2*a*–*c*). However, more proximally, in the supporting cartilage furrow, developing saw-teeth are not oriented in the lateral plane (figures 1*c*,*d* and 2*a*–*c*,*e*; electronic supplementary material, figure S2a–c). Although these orientations may appear random, their precise, gradual rotation laterally into position beneath roots of pre-existing saw-teeth is a constant feature. Newly developing saw-teeth are enclosed by, and protected between, the elongated, bilobed, divided roots of functional saw-teeth (providing the generic name). Because of this mode of saw-tooth development, in a fossil, there is potential for developmental data to be obtained, for comparison to oral dentitions.

To investigate the arrangement of functional teeth and the series of replacement teeth beneath them, we used specimen NHMUK PV P.73626, a partial but exceptionally well-articulated rostrum tip, with the smallest saw-teeth adjacent to the tip, and becoming larger caudally (figures 1*a*–*c* and 2*a*–*c*; electronic supplementary material, figure S1d). *In vivo* exposed arrowhead-shaped crowns are closely spaced and overlapping, producing the serrated blade with a regular alternate, flattened crown pattern. Virtual dissection of the roots exposed the mineral-dense, successively generated saw-tooth crowns in a strikingly regular space-filling arrangement (figures 1*b* and 2*a*). Developing and functional crowns form a close-packed arrangement with roots extending to the rostrum support cartilage (figures 1*a*–*e* and 2*a*,*b*). These virtual serial dissections also expose the newest developing saw-teeth (figures 1*b*,*c* and 2*a*). Saw-teeth of serially iterative, successive stages form within the rostrum saw-blade (figures 1*c*–*e* and 2*d*,*f*; electronic supplementary material, figure S1c) as a ‘cone within cone’ arrangement (ready-made saw-teeth numbered 3–6), sheltered within the roots (figure 1*d*,*e*; electronic supplementary material, movie; http://dx.doi.org/10.5519/0068733).

To inform the type of tissues comprising the saw-tooth blade, we examined thin sections of the entire blade, cut in the vertical plane (figure 1*f*–*l*; electronic supplementary material, figure S2a–f). These showed the newest saw-tooth crowns and the highly mineralized saw-tooth support cartilage forming the developmental furrow (figure 1*f*, tsc, t1, t2; electronic supplementary material, figures S1 and 2a–c). Attachment of the saw-tooth stack to the supporting (non-tesselated) cartilage is via extensive and numerous Sharpey's fibres (double arrow; figure 1*f*, *, tr, 1 L, sfb; electronic supplementary material, figures S1 and S2d,f). This is unusual in cartilage but relates to attachment of the substantial roots infilled with osteodentine, the latter easily identified by regular vascular canal spaces with radiating dentine tubules leading from them. This also fills saw-tooth crowns (relative to unfilled small, developing crowns) and the closely packed roots, enclosing developing teeth (figure 1*f*,*g*,*i*–*k*, od, tr-od; electronic supplementary material, figure S2a–c,e,f). This tissue substitutes for bone (absent in chondrichthyans), and with the fibrous attachment of the roots to the cartilage provides extra stability of the saw-tooth edge (double arrow, figure 1*f*,*g*,*l*), as well as substantial protection for the developing saw-teeth.

**Figure 3:**
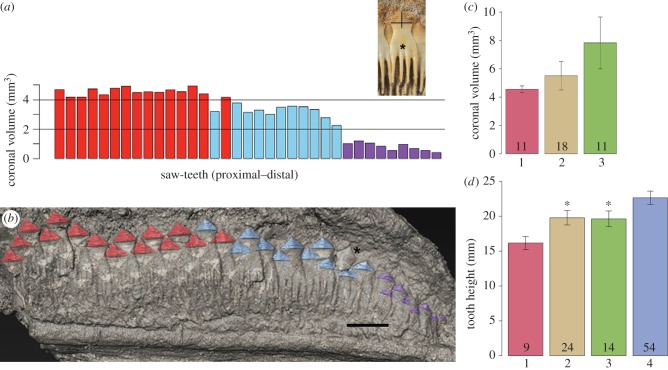
Comparative measurements of crown volume and saw-tooth height. *Schizorhiza stromeri* Maastrichtian (Latest Cretaceous) Oved Zem region, Morocco. (*a*,*b*) NHMUK PV P.73626, distal rostrum as in figure 1. (*a*) Graph shows volume of individual saw-tooth crowns, proximal to distal. Inset, individual saw-tooth, horizontal black line delineating coronal volume and vertical line showing height measurement. (*b*) Specimen with three colours equivalent to crowns measured in (*a*). (*c*) Mean coronal volume in NHMUK PV P.73626 (rostrum 1; also distal rostrum teeth—electronic supplementary material, figure S1d—excluding smallest teeth near addition site), Naturhistorisches Museum in Wien Inv.NR 1999z009/0001a (rostrum 2, also electronic supplementary material, figure S1a–c) and NHMUK PV P.73627 (middle rostrum 3, also electronic supplementary material, figure S1e,f), revealing a significant volumetric difference between rostral saw-teeth from these three regions (one-way ANOVA *F*_3,37_ = 23.8, *p*-value < 0.001). (*d*) Mean saw-tooth height in rostrum 1 (excluding smallest teeth near distal addition site), rostrum 2, rostrum 3 and complete rostrum 4 (NHMUK PV P.73625, excluding smallest teeth near distal site of rostrum growth; also electronic supplementary material, figure 1). Asterisks denote specimens not significantly different from one another. Individual *p*-values from the Tukey multiple comparisons of means available in the electronic supplementary material.

**Figure 4.**
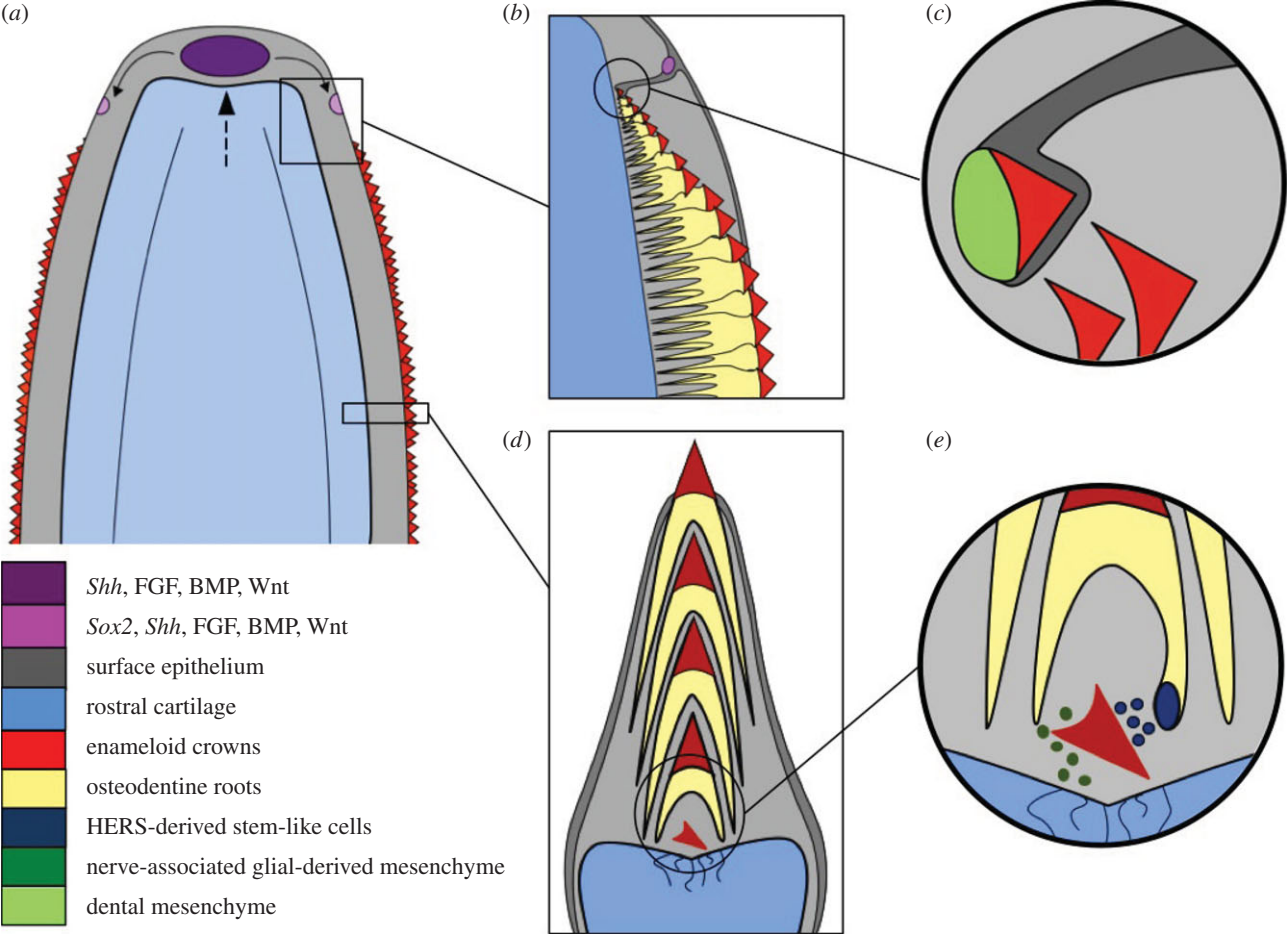
Schematic reconstruction of the developmental origins of saw-tooth addition and replacement. *Schizorhiza stromeri* (Maastrichtian (Latest Cretaceous) Oved Zem region, Morocco). Information is extrapolated from developmental studies in modern day taxa. (*a*) Distal (anterior) region of the saw-tooth rostrum showing a suggested midline symphyseal signalling centre (purple) that orders rostrum growth (blue, rostral cartilage) (dotted arrow) and provides space for initiating saw-tooth addition more proximally (pink, saw-tooth initiation sites). (*b*,*c*) Dorsal view of proposed saw-tooth addition site where interactions between skin epithelium (invaginating epithelial dental lamina) and underlying neural-crest-derived mesenchyme give rise to new saw-teeth—a process developmentally linked to rostrum extension. Epithelial invagination and odontogenic competency would have likely arisen from expression of known, conserved markers of tooth initiation including perhaps *Sox2*, *Shh* and members of the Wnt/β-catenin pathway. Subsequent saw-tooth development would have been guided by spatio-temporal expression of *Shh*, and members of the BMP, Wnt/β-catenin, FGF and Notch signalling pathways. (*d*) Transverse cross-section (based on micro-CT scans and soft tissue interpretation) through a saw-tooth replacement family, showing site of saw-tooth development above the highly vascularized cartilage furrow; growing root of the predecessor saw-tooth (root epithelial sheath HERS + dental papilla) would probably house competent cells responsible for tooth replacement (stem cell-mediated). (*e*) Close-up schematic of the cartilage furrow (in (*d*)), detailing hypothetical stem-like cellular interactions between epithelium derived from HERS-like cells (blue circles) with neural crest and nerve-associated glial-derived mesenchyme (dark green circles).

To determine the location of the rostrum growth centre, we compared numbers of saw-teeth per replacement file and their measurements. These showed that with rostrum growth, saw-teeth increase in number and size equally, away from the rostrum tip (figures 1*a*,*b*, 2*a* and 3; electronic supplementary material, figures S1d and 3a,b, arrowhead), which entirely lacks mineralized saw-tooth germs (figure 2*c*, white arrow; electronic supplementary material, figure S3b, asterisk). This region can be interpreted as the soft tissue growth centre for both cartilage and saw-teeth (figure 4). To determine whether saw-teeth were replaced, we compared crown volumes, showing that those near the rostrum tip are demonstrably smaller. These must be shed and replaced during growth, to reach the maximum saw-tooth size found along the rostrum (figure 3; electronic supplementary material).

We investigated how saw-teeth were regenerated and replaced, as tooth renewal is an important part of the control mechanism for oral teeth; we also examined whether this renewal always occurred at the same rate. We compared saw-teeth along the rostrum, from the proximal chondrocranium to the distal rostrum tip, with proximal saw-teeth significantly larger (figure 3*a*,*b*). As noted, multiple new saw-teeth develop close to the rostrum tip (figures 1*a*–*c*, 2*a*–*c* and 3*b*); by comparison, there are markedly fewer new saw-teeth developing in the proximal or middle rostrum, where form and size stabilizes (figure 2*d*–*f*, red crowns). This indicates that replacement had slowed in the more proximal, older regions, with the greater rate of saw-tooth renewal associated with the rostrum growth centre near the tip.

We sought evidence for control of timing and spatial organization of saw-teeth that would be produced by a precise, genetic regulatory developmental mechanism, as within oral teeth [13,14]. We found that the gradual, precise rotation of new saw-tooth crowns into position beneath roots of pre-existing saw-teeth is a frequent feature of the growth region (figures 1*a*–*e* and 2*a*–*f*; electronic supplementary material, figure S2a–c). A model of regular spatio-temporal rotation of the saw-tooth germs is proposed from observations of the first mineralized crowns, with different mineral densities, allowing a temporal developmental pattern to be identified (figure 1*c*,*d*). Crowns with increasing densities are numbered in developmental order to illustrate the gradual steps of their rotation (figures 1*c*–*e*, 1–6, and 2*b*,*c*, 1–5). From the earliest crowns, lying against the cartilage (laterally flattened) pointing caudally, they first rotate 90° into the lateral plane (dorsoventrally flattened) and then continue to rotate through 90° relative to the lateral saw edge (figure 1*c*,*d*, 4–6) until they lie below the previous saw-tooth's roots (4), prior to forming roots. These observations of mineralized crowns developing with controlled, multiple rotations in *Schizorhiza* are different from any chondrichthyan reported. However, the second rotation is similar to the movement in sharks that developed teeth make to come into the functional position at the jaw margins (also the rotation of the saw-teeth in sawsharks Pristiophoridae [7,10]), and to tooth rotation in a variety of teleost fishes (e.g. Elopomorpha, Characiformes), where regenerated teeth rotate through 90° into position, to replace functional teeth along the jaw [15]. Also, the development and replacement of saw-teeth positioned directly beneath the roots of functional ones (made of osteodentine, a bone substitute) shows more similarities to oral tooth replacement in some osteichthyans with intraosseus tooth development [15–17] than to chondrichthyans with replacement teeth in soft tissue within the dental lamina. This is presumed to be true for oral dentitions of *Schizorhiza*, but no articulated jaws with teeth are known.

## 4. Discussion

We have presented data for *Schizorhiza* showing how the individual developmental module (saw-tooth) is ordered into a structural pattern along the rostrum saw in two ways: initiation of saw-tooth files at the rostrum growth centre, linked with establishment of replacement saw-teeth during growth, then in maintenance of the saw edge through regeneration. These parameters define the oral dentition in chondrichthyans but tied to and dependent on a dental lamina. Saw-tooth development in *Schizorhiza* as well as in others with elongated rostra [10] can test hypotheses that oral teeth can be derived from modification of external skin denticles, as proposed in canonical theories of tooth evolution.

We have demonstrated that *Schizorhiza* preserves an unusually high degree of developmental data for a fossil, in building the rostrum saw through saw-tooth addition, with growth and renewal, larger saw-teeth replacing small. We suggest there were two important sites for saw-tooth initiation: (i) the rostrum tip, as a symphyseal signalling centre, regulating initiation to the left and right of the tip, which otherwise remains free of saw-teeth; (ii) more posteriorly along the cartilage furrow, regulating regeneration below the saw-tooth stacks (figures 1–4). As shown in our interpretive model in figure 4, the rostrum tip signalling centre would express conserved gene markers linked to cartilage growth and extension and odontogenesis, including members of the Hedgehog, Bmp, Fgf and Wnt/*β*-catenin signalling pathways [18]. The rostrum cartilage furrow with odontogenic competence for continued saw-tooth replacement was rich in a vascular supply from rostrum cartilage blood vessels [7], supplying saw-tooth root growth (figure 1*f*, bv). Here, vascularized, innervated tissue could provide multipotent stem cells (perivascular, neural derivatives) contributing to saw-tooth development and renewal (figure 4*d*), comparable with those stem cells demonstrated to contribute to development, growth and renewal of mammalian teeth [19]. Additionally, the outer dental epithelial cells associated with the extended saw-tooth root system could act as a source of epithelial and mesenchymal stem cells similar to those of oral dentitions, for saw-tooth regeneration (Hertwig's root sheath, HERS [20]; figure 4*e*). We suggest a further potential neurovascular-based origin for signals at the rostrum tip growth centre (derived from nerve-associated glial cells, NAGCs [19,21]) directing and maintaining stem cell activity for continued growth with saw-tooth renewal. Notably, the rostrum in extant chondrichthyans is rich with sensory ampullae; these could be a source of NAGCs, linked to the proposed symphyseal signalling centre. Although these growth centres are identified in the fossil *Schizorhiza*, our reconstructions and interpretations can be tested using modern sharks and rays as developmental models (for example, the sawshark).

We conclude that neither *Schizorhiza* nor other chondrichthyan taxa with a saw-tooth rostrum exactly replicate the developmental organization and structural arrangement of teeth in the oral dentition [10,22]. In *Schizorhiza*, developmental rotation of saw-teeth is unique, while the position of successive, replacement saw-teeth directly below one another is more similar to the osteichthyan dentition [11,15,23] than to the replacement of oral teeth of any shark or ray. Therefore, we agree that rostrum saw-teeth are highly modified dermal denticles [24,25], but propose that they have diversified after co-option from a regional, symphyseal-based system, such as organized skin denticles located at, or near, the rostrum tip (M.M.S. & Z.J. 2015, personal observation). This is convergent with the oral dentition of sharks and rays. The capacity for organized succession and renewal demonstrated in *Schizorhiza* informs the process of skin denticle diversification through developmental plasticity, representing a true paradigm for patterning ‘teeth outside the mouth’, but distinct from ‘teeth inside the mouth’, associated with articulating jaws [3].

## Supplementary Material

Figure 1. Figure 2. Figure 3

## Data accessibility

Data can be accessed via the NHM Data Portal (http://data.nhm.ac.uk; http://dx.doi.org/10.5519/0068733; http://dx.doi.org/10.5519/0091934).
