# Supplementary material for: Early development of rostrum saw-teeth in a fossil ray tests classical theories of the evolution of vertebrate dentitions
Source: Proc Biol Sci. 2015 Oct 7;282(1816):20151628. doi: 10.1098/rspb.2015.1628 (PMC4614774; doi:10.1098/rspb.2015.1628)
Supplement: Figure 1. Figure 2. Figure 3 [file rspb20151628supp1.docx]

**Supplementary Material**

**Early development of rostrum saw-teeth in a fossil ray tests classical theories of the evolution of vertebrate dentitions**

Moya Meredith Smith^1,2^, Alex Riley^1^, Gareth J. Fraser^3^, Charlie Underwood^4^, Monique Welten^1,†^, Jűrgen Kriwet^5^, Cathrin Pfaff^5^, Zerina Johanson^1,*^

*Saw-tooth size variation*

Using the Aviso segmentation tool, we measured the volume of the arrowhead-shaped saw-tooth crowns from their tips down to their widest point. Our data from NHMUK PV P.73626 shows rostral saw-tooth size increased proximally from the distal rostrum tip, with large saw-teeth of over 4mm^3^ (red most proximal, Fig. 3A, B) then distally smaller, saw-teeth between 2mm^3^ and 4mm^3^ (sky blue, Fig. 3A, B), and smallest saw-teeth closest to the distal end being, less than 2mm^3^ (purple, Fig. 3A, B). Such an increase in saw-tooth size at the rostrum tip strongly suggests that cyclical rostral saw-tooth replacement results in larger saw-tooth sizes with age, as these smaller saw-teeth are lost during growth. Previously described *Schizorhiza* fossils were said to show a “slight increase in tooth size from the distal to more proximal end of the rostrum” [8] our measurements of saw tooth height (from crown tip to start of root bifurcation), including along an articulated, near-complete rostrum (Fig. 3D) show a significant increase in height with rostrum growth. This, along with observations on saw-tooth size and numbers of very small teeth forming into tooth sets for replacement at the most distal part, suggests that the tip represents the region of rostrum growth (Fig. 4A).

*Saw-teeth successional development*

We can explain the apparent disorder of the newest saw-teeth in the developmental furrow along the rostrum by rotation of the developing saw-tooth germs during life, rather than post-mortem changes (e.g., Fig. 1B, C). This movement forms a double 90º rotation from flat against the support cartilage (1), to flat within the dorso-ventral plane (2), then gradually the crown tip direction changes from caudal to lateral (3, 4) until normal to the functional saw-tooth edge (5, 6; Figs. 1C, D; 2B, C).

The earliest observed saw-tooth developmental stages consist of hollow crowns (Figs. 1E (3), F, t1, 2B, magenta, ESM Fig. 2A-C). Saw-tooth rotation can also be visualized in sections through the saw-edge, showing the developing saw-tooth crown lying almost flat against the support cartilage (Fig. 1F; ESM Fig. 2A-C), as a hollow shell of enameloid and a thin layer of pallial dentine. The next saw-tooth in the developmental series lies below the roots of several saw-teeth also rotating and not yet in the functional position, with a thicker layer of dentine, then nearly lateral when the roots are growing (Figs. 1F, t2; 2B, saw-teeth in series coloured magenta, green, orange, pink, blue, red; Supplementary Fig. 2A-C, E). This rotation is associated with the initial development of osteodentine, within the crown and at the apical region of the root (Fig. 1G, I, K; ESM Fig. 2A-C). Continued root development is accompanied by a migration of the saw-tooth crown away from the lateral surface of the rostral cartilage as additional saw-teeth start to develop along the lateral groove in the rostral cartilage (e.g., Fig. 1C).

*Saw-tooth replacement with age*

Further evidence for cyclical rostral saw-tooth replacement is provided by our comparative measurements between four *Schizorhiza* fossils. Our coronal, volumetric measurements of rostral saw-tooth crowns reveal that there is a significant difference between the largest rostral saw-teeth of NHMUK PV P.73626 and those of Naturhistorisches Museum in Wien Inv.NR 1999z009/0001a and NHMUK PV P.73627 (Fig. 3C); statistical significance was calculated using a Tukey multiple comparisons of means test (P.73627-P.73626: p-value < 0.001; NMWInv.NR 1999z009/0001a-P.73626; p = 0.0376; NMWInv.NR 1999z009/0001a-P.73627, p-value < 0.001). These observations are supported by the total length of undamaged rostral teeth between specimens, with those of NMWInv.NR 1999z009/0001a and NHMUK PV P.73627 being significantly longer than saw-teeth of NHMUK PV P.73626, and those of NHMUK PV P.73625 being significantly longer than all other specimens (Fig. 3D); statistical significance was calculated using a Tukey multiple comparisons of means test (P.73627- P.73626, p-value < 0.001; P.73625-P.73626, p-value < 0.001, NMWInv.NR 1999z009/0001a-P.73626; P.73625-P.73627, p-value < 0.001; NMWInv.NR 1999z009/0001a-P.73625, p-value < 0.001). We found no difference in saw-tooth height between NMWInv.NR 1999z009/0001a and P.73627 (Tukey: p-value = 0.963), as indicated by asterisks above bars. Such marked changes in saw-tooth size between specimens suggest that cyclical replacement must occur with each successor saw-tooth being slightly larger than its predecessor. Together with regular increments in tooth size, variation of coronal volumes also increases (0.058mm^3^, in NHMUK PV P.73626, 1.01^3^ in NMWInv.NR 1999z009/0001a, and 3.33mm^3^ in NHMUK PV P.73627).

*Saw-tooth tissue histology*

We also compared virtual and real sections, vertical to the saw-tooth blade, to demonstrate the organization of the stack of saw-teeth below one functional tooth, each saw-tooth sheltered within the roots of the older saw-tooth in the stack (Fig. 1E; ESM Figs. 2A-C, E, 3C,). To inform the type of tissues that comprised the saw-tooth blade we made photomicrographs of the polished surface and thin sections (Fig. 1F-L, ESM Fig. 2) in the same vertical plane to show both the developmental furrow, with new saw-tooth crowns, and attachment of the saw-tooth stack to the support cartilage of the extended rostrum (Fig. 1A, double arrow; 1E, *, tr, tsc; 1F, t1, ESM Fig. 2).

The tissue of the saw-tooth crown tip forms a sharp point (Fig. 1E-H; ESM Fig. 2A, C, F), composed of two types of dentine: central osteodentine filling the pulp chamber, surrounded by thick pallial dentine with many branching dentine tubules (Fig. 1G, I, ESM Fig. 2E, F). These lead into fine tubules of the thinner enameloid (Fig. 1K). Enameloid crystallite direction is normal to the surface of the crown (blue or yellow colour, Fig. 1H, I). The central osteodentine has many pulp canals, assumed to be cellular and vascular, with variable direction of crystal-fibre bundles depending on the formative layers (Fig. 1J, K). Pulp canals at the centre of the denteones branch many times and finer tubules lead into and through all of the dentine into the pallial zone and the enamleloid (ESM Fig. 2E, F). Osteodentine fills all crowns (except of the earliest saw-teeth, Fig. 1F, t1, fig. SI2A, C), and all roots that pack around the developing teeth, enclosing them and acting as support for the saw-tooth edge (double arrow, Fig. 1F, G; Suppl. Info. Fig. 2A, C).

As tissue arrangement is part of the developmental process of all teeth, the observation that osteodentine fills all crowns (except of the earliest saw-tooth germs, Fig. 1F, t1) and all roots that pack around the developing saw-teeth, enclosing them, ensures maximum support for the saw-tooth edge (double arrow, Fig. 1F, G). This tissue acts as a substitute for bone, normally absent in all chondrichthyans, so that modification of the external rostrum placoid denticles into saw-teeth with this basal bone substitute, together with the fibrous attachment of the roots to the cartilage support tissue ensures extra stability of the saw-tooth edge (Fig. 1F, G, L). The saw-tooth rostrum support cartilage is highly mineralized (ESM Fig. 2A-C) with a fibre-bundle texture alongside many vascular canals, closely opposed to similar fibres in the roots (Fig. 1F, L, double arrow). Polarised light with determination of the birefringence sign shows crystal-fibre-bundle orientation in the cartilage and their predicted continuity across a soft tissue gap (asterix, Fig. 1F, L, ESM Fig. 2B, C, D, F) with those of the saw-tooth roots (blue colour, +45 orientation, Fig. 1L, double arrow). Direction of crystal fibres in the attachment zone indicates that a fibrous attachment (Sharpey’s fibres) joins the roots of saw-tooth file to the mineralized support cartilage of the rostrum (Fig. 1L, double arrow for root width, Sharpey’s fibres of the cartilage, ESM Fig. 2D, F).

**Systematic considerations**

*Schizorhiza stromeri* is member of an extinct clade of saw-tooth rays, Sclerorhynchoidei, comprising circa 25 genera within two families, Sclerorhynchidae and Ptychotrygonidae [8, 12, 26]. Most taxa are only known by their distinct isolated rostrum teeth, and occasionally, oral teeth, whereas partial or complete specimens were mainly recovered from Cenomanian (ca. 96 million years) and Santonian (ca. 85 million years) deposits of Lebanon [12, 27] resulting in different or incomplete data sets for phylogenetic reconstructions. Consequently, the inter- and intrarelationships of sclerorhynchids and sclerorhynchoids are still controversial [11, 12, 28].

Morphological characters identifying sclerorhynchids as a monophyletic group within rays and skates (Batoidea) are mainly related to rostral teeth and their replacement pattern in few completely articulated specimens [28]. These characters, however, might be plesiomorphic when compared with saw-tooth sharks [29]. Systematic separation of taxa within sclerorhynchids also is difficult because rostral teeth are the only skeletal elements known from all members.

Rostral tooth replacement in almost all sclerorhynchids occurs as newly formed teeth erupting close to functional teeth [28]. This is unlike the condition seen in *Schizorhiza stromeri*, in which numerous replacement teeth are uniquely stacked ‘cone within cone’ below functional teeth. This battery-like overlapping of rostral teeth in *Schizorhiza* differentiates it easily from other sclerorhynchids and identifies it as representative of a distinct, monogeneric subfamily, Schizorhizinae [28]*.* Differences in tooth replacement patterns and rostral tooth morphologies imply that Sclerorhynchidae and probably Sclerorhynchoidea might not be monophyletic conversely to previous assumptions [11, 12, 28, 30].

The histology of these rostral saw-teeth might provide some additional information. *Schizorhiza* shares with other larger saw-tooth rays such as *Onchosaurus* and *Pucapristis* rostral teeth with a core of osteodentine. Contrary to this, the crown of the rostral teeth in other sclerorhynchids, of which 15 genera have been histologically studied up to now, consists of orthodentine with a pulp cavity. The different histologies indicate that at least two different monophyletic groups might exist within Sclerorhynchidae pending further analyses of those sclerorhynchids in which the rostral tooth histology remains unknown, but now reported here in *Schizorhiza*.

**References**

26. Kriwet J, Nunn EV, Klug S. 2009. Neoselachians (Chondrichthyes, Elasmobranchii) from the Lower and lower Upper Cretaceous of north-eastern Spain. *Zool. J. Linn. Soc. Lond.* **155**, 316-347.

27. Forey PL, Yi L, Patterson C, Davies CE. 2003. Fossil fishes from the Cenomanian (Upper Cretaceous) of Namoura, Lebanon. *J. Syst. Palaeontol*. **1**, 227-330.

28. Kriwet J. 2004 in *Mesozoic Fishes 3 - Systematics, Paleoenvironments And Biodiversity* (eds Arratia, G. & Tintoria, A.) 57-73 (Verlag Dr. Pfeil, Munich, Germany).

29. Arambourg, C. 1940. Le groupe des Ganopristinés. *B. Soc. Geol. Fr.* **10**, 127-147.

**
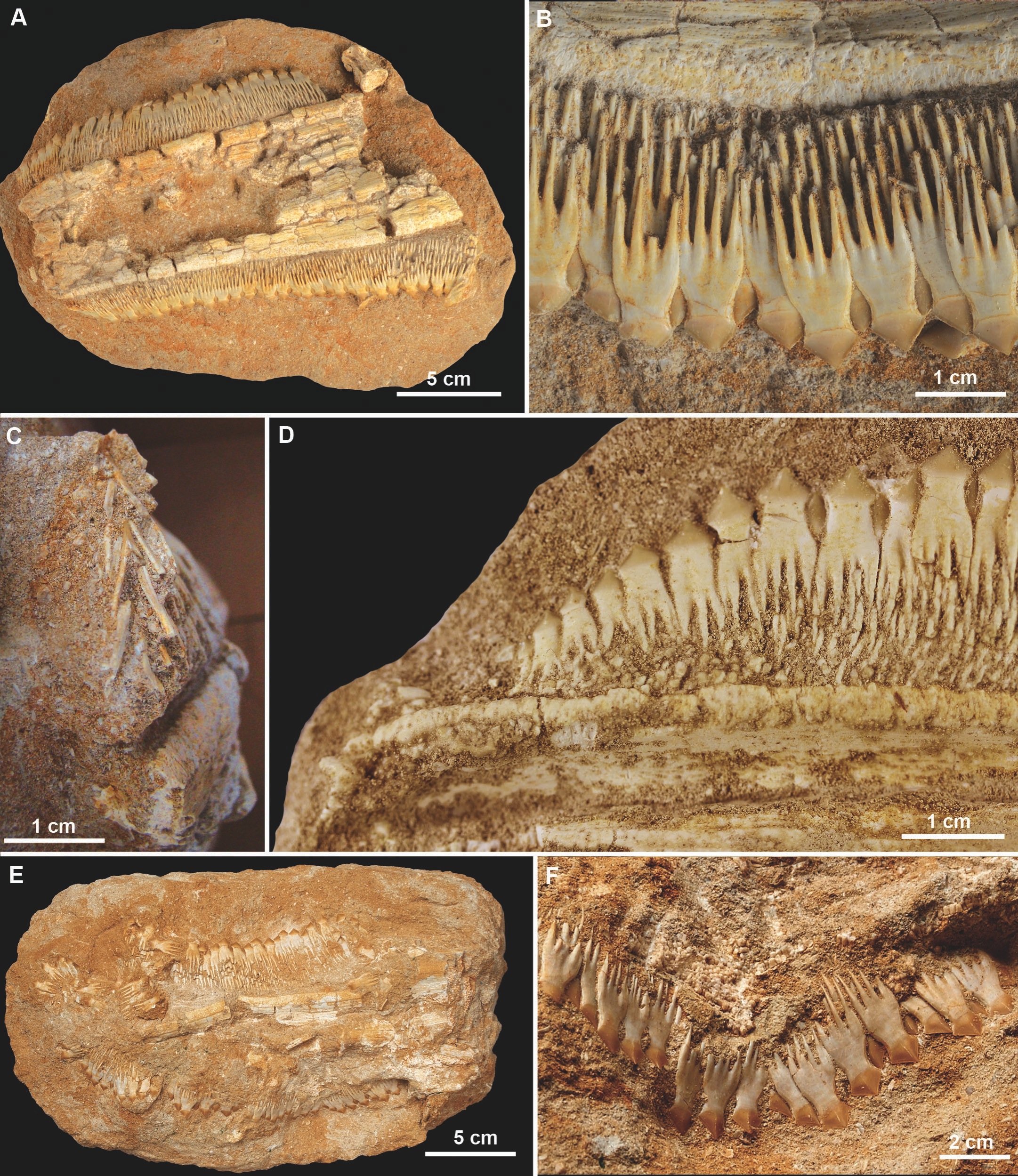
**

**Fig. 1 Three articulated rostra with detail of saw-teeth at forming distal rostrum tip.**

*Schizorhiza stromeri* Maastrichtian (Latest Cretaceous) Khourubga region, Morocco. *(A-E)* Naturhistorisches Museum in Wien Inv.NR 1999z009/0001a. A, incomplete mid-portion of rostrum with saw-tooth files. *(B)* closeup of saw-teeth and cartilaginous rostrum showing arrowhead shaped crowns and roots divided distally into four prongs. *(C)* broken edge of specimen showing characteristic tooth files in section, with distinct ‘cone within cone’ stacking along the cartilaginous rostrum. *(D)* NHMUK PV P.73626, macrophotograph. *(E, F)* NHMUK PV P.73627, *(E)* incomplete mid-portion of rostrum with saw-tooth files, *(F)* closeup of saw-teeth and cartilaginous rostrum showing arrowhead shaped crowns and roots divided distally.

**
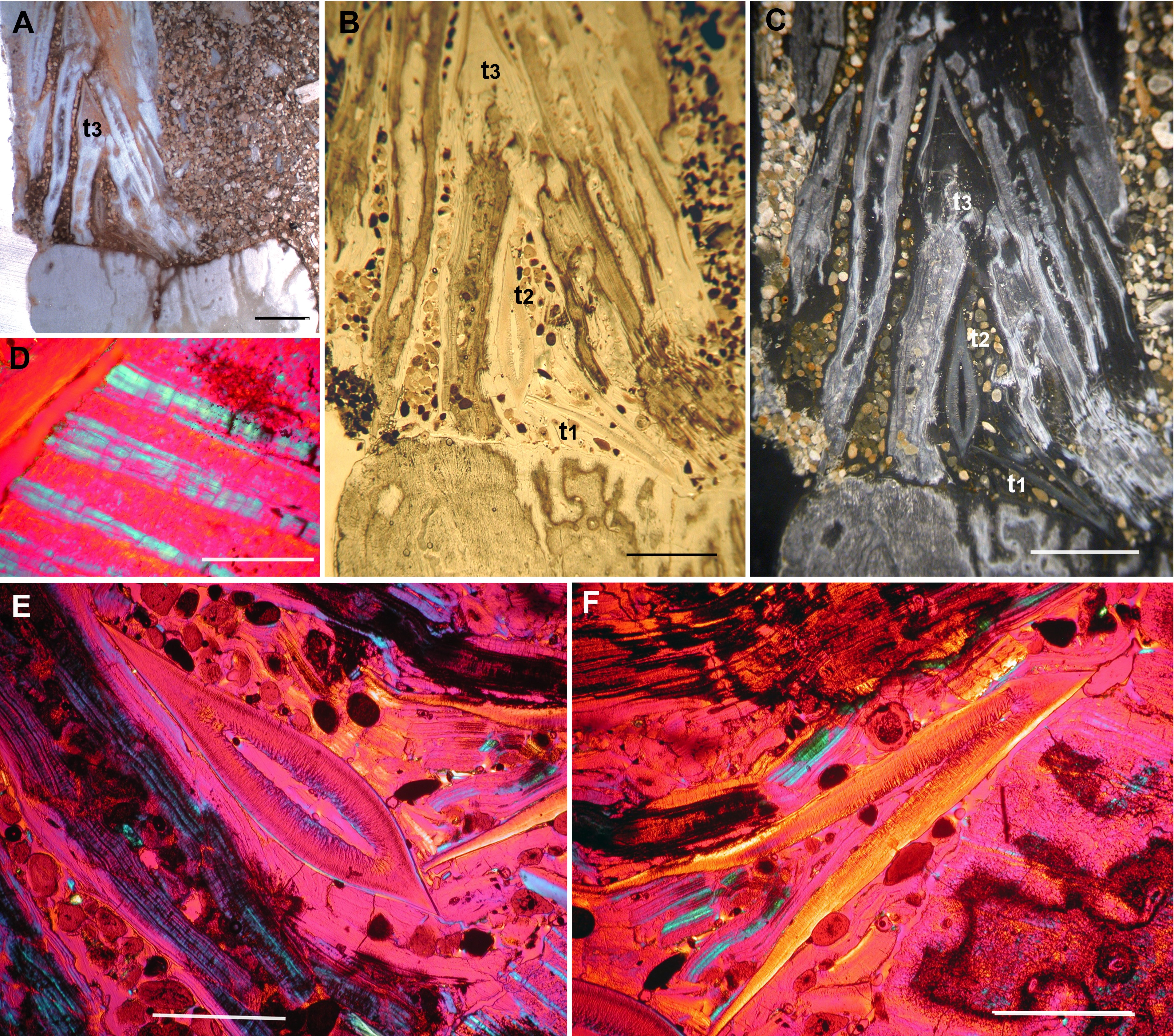
**

**Figure 2 Histology of vertical section through developing region of saw-edge.**

*Schizorhiza* *stromeri* Maastrichtian (Latest Cretaceous) Khourubga region, Morocco. *(A-F)* NHMUK PV P.73626. *(A)* surface of section showing saw-tooth file, with distinctive ‘cone within cone’ stacking of newer saw-teeth between roots of the previous saw-tooth, above the furrow in the supporting cartilage (t^3^ is the same laterally directed new tooth with roots in *(A-C)*); all show osteodentine in crown and roots; lateral cartilage with many assumed neurovascular canals. *(B, C)* smallest new teeth with one caudally directed and the other horizontal (t^1^, t^2^, below t^3^; both very translucent, equates with high mineral density as in crown of t^3^). *(D-F)* crossed polars with gypsum plate inserted to show sign of birefringence of mineral (HAP) crystals, hence direction of organic matrix of collagen fibres; *(D)* field immediately below these saw-tooth roots, thick and numerous attachment fibres of Sharpey into highly mineralized support cartilage (soft tissue gap top left); *(E, F)* fields of two newest teeth (t^1^, t^2^, comparable with those in Figs. 1, 2)).

**
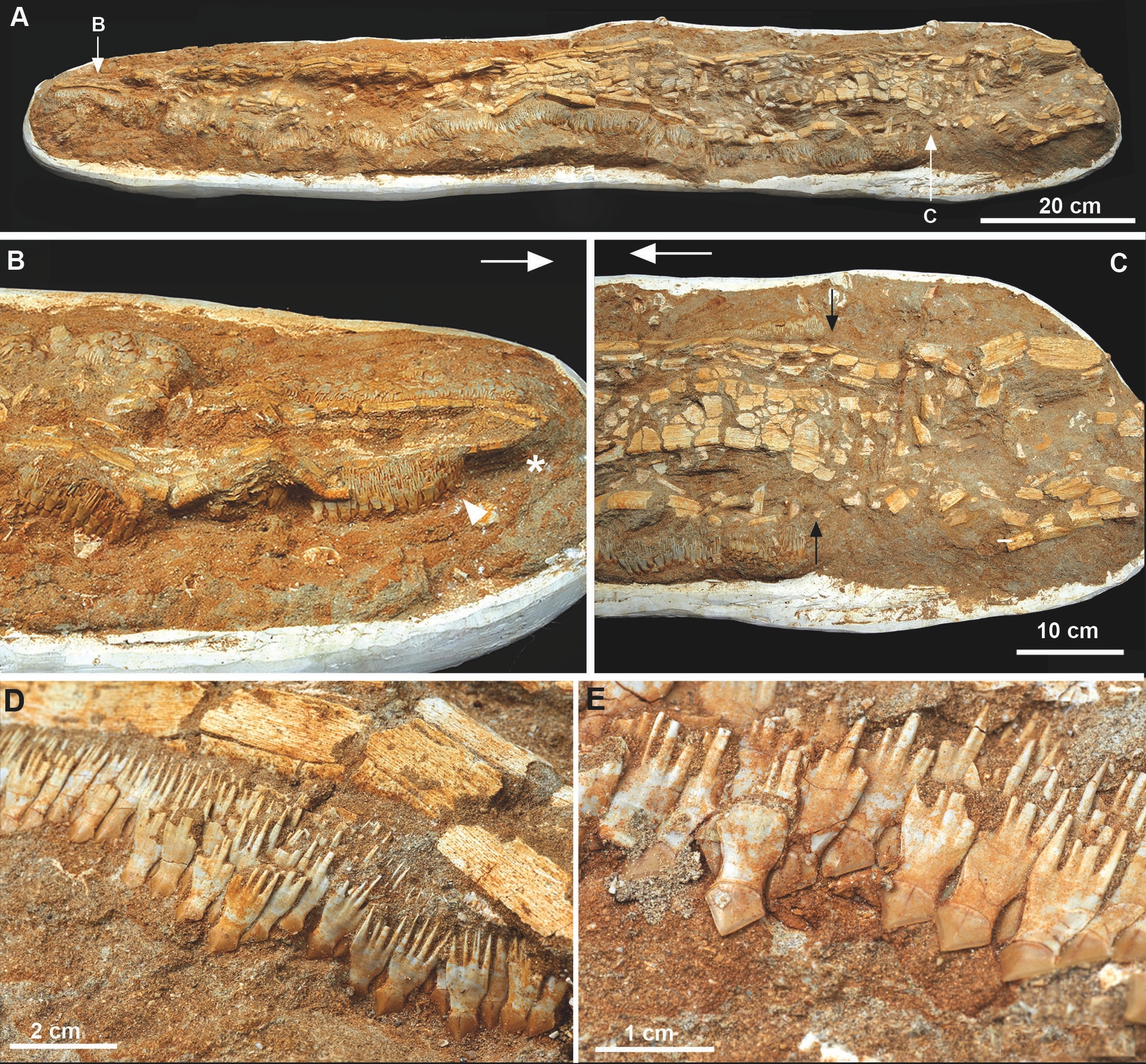
**

**Fig. 3 Complete articulated rostrum with saw-teeth of rostrum tip and base.**

*Schizorhiza stromeri* Maastrichtian (Latest Cretaceous) Khourubga region, Morocco. *(A-E)* NHMUK PV P.73625. *(A)* nearly complete rostrum including tip *(B)*, extending proximally *(C)*, most proximal part of rostrum has been lost. *(B)* rostrum tip with distinctly smaller saw-teeth (arrowhead), with saw-teeth absent at tip itself (asterisk), white arrow indicates anterior. ***(****C)* more proximal section showing point where saw-tooth files begin (small black arrows), rostrum lacking saw-teeth continues proximally, white arrow indicates anterior. *(D, E)* closeups of saw-teeth and cartilaginous rostrum, showing arrowhead-shaped crowns covered in enameloid, mineralized roots with some variation in thickness of the separate prongs.

**Supplementary Movie**

This shows the segmented stack of saw-teeth from Figure 1C, D in relation to all other saw-teeth and can be accessed from the NHM Data Portal ([http://dx.doi.org/10.5519/0068733](http://dx.doi.org/10.5519/0068733)).
